# A genome-wide scan maps a novel autosomal dominant juvenile-onset open-angle glaucoma locus to 2p15-16

**Published:** 2008-04-18

**Authors:** Ying Lin, Ting Liu, Jing Li, Jiyun Yang, Qiong Du, Junfang Wang, Yang Yang, Xiaoqi Liu, Yuanfu Fan, Fang Lu, Yilian Chen, Yonghong Pu, Kang Zhang, Xiangge He, Zhenglin Yang

**Affiliations:** 1 Human Molecular Biology and Genetics, Sichuan Academy of Medical Sciences and Sichuan Provincial People’s Hospital, Sichuan, China; 2 Department of Ophthalmology, Daping Hospital, the Third Military University of Medical Sciences, Chongqing, China; 3 Department of Ophthalmology, Sichuan Academy of Medical Sciences & Sichuan Provincial People’s Hospital, Sichuan, China; 4 Zhongxian County Hospital, Chongqing, China; 5 Department of Laboratory Medicine, Chengdu University of Traditional Chinese Medicine, Sichuan, China; 6 Department of Ophthalmology and Visual Sciences, Program in Human Molecular Biology & Genetics, Eccles Institute of Human Genetics, University of Utah Health Sciences Center, Salt Lake City, UT

## Abstract

**Purpose:**

To study the clinical features and to perform genetic linkage study in two large Chinese families with autosomal dominant juvenile-onset primary open-angle glaucoma (POAG).

**Methods:**

Eighteen members of one Chinese family and 25 members of a second Chinese family with juvenile-onset primary open-angle glaucoma (POAG) were investigated. Thirteen members in one family and 14 members in the second family were diagnosed with juvenile-onset POAG. A genome-wide linkage scan was performed on one family using 411 short tandem repeat (STR) markers. Subsequent fine mapping was performed in the two study families using a modified fluorescent labeled M13 primer method.

**Results:**

A whole genome-wide scan in one family showed linkage to chromosome 2p15-p16 with a two-point maximum LOD score of 5.01 at θ=0 between the disease phenotype and STR marker D2S337. The second family was also mapped to the same locus with a two-point maximum LOD score of 6.30 at θ=0 for D2S378. Haplotype analysis in these two families demonstrated that they shared the same disease haplotype, suggesting they have inherited the mutation from a common founder. The maximum LOD scores were 8.93 at θ=0 for D2S378 and 9.9 at θ=0 for D2S337 when the two families were combined for analysis. The disease interval for these two families was localized to 9.2 cM or 13.3 Mb between D2S123 and D2S2397. There are 42 known genes/transcripts within the interval. Five of these genes were sequenced, and no disease-causing mutation was identified in either family.

**Conclusions:**

This novel juvenile-onset POAG locus on chromosome 2p15–16 is overlapped by the Glaucoma 1, open angle, H (GLC1H) locus for adult-onset POAG. Eventual identification of the disease-causing gene will provide insights into the pathogenesis of POAG.

## Introduction

Glaucoma is a leading cause of blindness in the world [1]. The disease causes irreversible, characteristic visual field loss and optic nerve damage, usually associated with elevated intraocular pressure (IOP). Glaucoma is a heterogeneous group of optic neuropathies that can be divided into congenital, juvenile-onset, and adult-onset categories, and it can be inherited as a Mendelian autosomal-dominant, an autosomal-recessive trait, or a complex multifactorial trait. Regarding the Mendelian inherited glaucoma, congenital is only inherited as autosomal recessive. Juvenile-onset and adult-onset glaucoma are inherited as autosomal dominant. The majority of glaucoma cases (60%–70%) are associated with a normal-appearing trabecular meshwork, a visual field loss, and a frequently elevated intraocular pressure (IOP). This presentation of glaucoma is termed primary open-angle glaucoma (POAG) [2]. The affected POAG patients may maintain useful sight if the disease is treated before significant damage to the optic nerve occurs. Therefore, early diagnosis is critical for vision loss prevention. Juvenile-onset open-angle glaucoma is a subset of POAG that appears earlier in life, usually before 40 years old, and is inherited in an autosomal dominant manner [3]. Seven loci for adult-onset autosomal dominant POAG have been mapped, including GLC1A (*Myocilin*, *MYOC*; 1q23), GLC1B (2cen-2q13), GLC1C (3q21–24), GLC1D (8q23), GLC1G (*WD repeat-containing protein 36*, *WDR36*) (5p22), GLC1F (7q35-q36), and GLC1H (2p15-p16) [3-8]. For juvenile-onset autosomal dominant POAG, only five loci, including GLC1A (*MYOC*) (1q23), GLC1J (9q22), GLC1K (20q12), 5q22.1-q32, and 15q22-q24, have been mapped, and only one gene (*MYOC*) has been identified [4,9-13]. Mapping and identifying new loci and genes for juvenile-onset POAG will contribute to the understanding of the pathogenesis of glaucoma. Here, we report that two juvenile-onset families map to the 2p15-p16 region.

**Figure 1:**
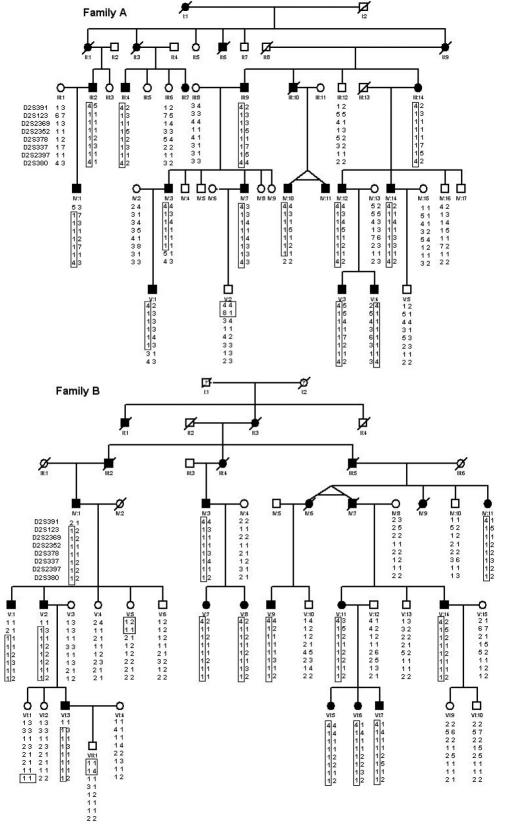
Pedigrees of two Chinese families with juvenile-onset primary open-angle glaucoma and genotype/haplotype data of eight short tandem repeat markers from the2p12-p16 region. The square symbol indicates male; the circle symbol denotes female; the filled symbol represents the affected family member with POAG; and the slashed symbol denotes that the family member is deceased. A phase-known disease haplotype in each family is indicated by a box.

## Methods

### Study subjects

This project was approved by the Institutional Review Board of Sichuan Academy of Medical Sciences and Sichuan Provincial People’s Hospital, Chengdu, Sichuan, China and by the Institutional Review Board of Daping Hospital of the Third Military University of Medical Sciences, Chongqing, China. This study included two large Chinese families. Eighteen family members were enrolled in the first family (Family A) and 25 members were enrolled in the second family (Family B; Figure 1). All family members underwent ophthalmic examination including visual acuity testing, tonometry, gonioscopy, and visual field testing. Clinical diagnosis was based on IOP, vision field loss, angle appearance, and optical disc appearance by the ophthalmologist specializing in glaucoma from Sichuan Academy of Medical Sciences and Sichuan Provincial People’s Hospital and Daping Hospital of the Third Military University of Medical Sciences. The patient was diagnosed with POAG using the following four criteria: optical damage (cup/disc ratio >0.5), visual field loss found by a Humphrey perimetry test, open-angle appearance by gonioscopy, and an IOP equal to or higher than 22 mmHg while not on medication.

### Genotyping and linkage analysis

Blood was collected by venepuncture, and genomic DNA was isolated from the samples using a PUREGENE blood kit from Gentra Systems (Biocompare Inc., San Francisco, CA). The known loci related to glaucoma, including GLC1A (1q23, *MYOC*), GLC3B (2p36), GLC3A (2p21, *cytochrome P450*, *subfamily I*, *polypeptide 1*, *CYP1B1*), GLC1B (2cent-2q13), GLC1C (3q21–24), *RIEG1* (4q25, *paired-like homeodomain transcription factor 2*, *PITX2*), GLC1G (5q22, *WD repeat domain 36*, *WDR36*), IRID1 (6p25, *forkhead box C1*, *FOXC1*), GLC1F (7q35), GPDS1 (7q35-q36), GLC1D (8q23), GLC1J (9q22), NPS (9q34, *LIM homeobox transcription factor 1*, *LMX1B*), GLC1E (10p15-p14, *optineurin*, *OPTN*), NNO1 (11p), AN2 (11p13, *paired box gene 6*, *PAX6*), VMD2 (11q12), MFRP (11q23), RIEG2 (13q14), GLC1L (15q11-q13), GLC1K (20p12), 5q22.1-q32, and 15q22-q24 [11], were examined by genotyping and linkage analysis for both families. A genome wide linkage scan was performed in Family A using ABI Linkage Mapping Set v2.5, which contained 411 short tandem repeat markers and True Allele PCR Premix (ABI, Foster City, CA) according to the manufacturer’s instructions. The amplified polymerase chain reaction (PCR) products were loaded on to the ABI 3100 Genetic Analyzer (ABI, Foster City, CA). We used the MLINK of the LINKAGE program to calculate two-point LOD scores (v.5.1; Human Genome Mapping Project Resources Center, Cambridge, UK) [14,15]. An autosomal dominant mode of inheritance with full penetrance and a disease allele frequency of 0.0001 were assumed in the calculations. For fine mapping, additional short tandem repeat (STR) markers were chosen from the Marshfield database and used for genotyping using a modified fluorescent labeled M13 primer method [16].

**Table 1:** Clinical information of the two Chinese families with primary open-angle glaucoma.

**Patients**	**Age/ age at diagnosis (years)**	**Highest IOP, mmHg**		**Visual acuity**		**Visual field loss**	**Cup-disc ratio**		**Treatment**
		**OS**	**OD**	**OS**	**OD**		**OS**	**OD**	
**Family A**									
III-2	70/25	69	>70	NLP	NLP	Yes	1	1	None
III-4	65/29	67	>70	NLP	NLP	Yes	1	1	Levobunolol administered
III-9	63/30	45	50	NLP	NLP	Yes	1	1	Levobunolol adminstered
III-14	63/50	58	>70	NLP	NLP	Yes	1	1	None
IV-1	36/31	24	27	20/70	20/50	Yes	0.8	0.7	Trabeculectomy
IV-3	39/19	41	>70	NLP	NLP	Yes	1	1	Trabeculectomy
IV-7	33/18	>70	>70	NLP	NLP	Yes	1	1	Levobunolol adminstered and Trabeculectomy
IV-10	29/20	21	22	20/30	20/30	Yes	0.6	0.3	None
IV-12	39/15	NA	>70	NA	LP	Yes	NA	1	Levobunolol administered and os removed
IV-14	32/25	69	>70	NLP	NLP	Yes	1	1	Traditional Chinese medicine administer
V-1	16/16	18	22	20/200	20/200	Yes	0.3	0.6	Timolol administered
V-3	14/14	28	25	20/100	20/70	Yes	0.3	0.5	Timolol administered
V-4	15/15	23	24	20/100	20/70	Yes	0.3	0.3	None
**Family B**									
IV-1	83/35	67	70	NLP	NLP	Yes	1	1	None
IV-3	56/26	30	>70	NLP	LP	Yes	1	1	Trabeculoplasty
IV-11	61/45	23	24	NLP	NLP	Yes	1	NA	None
V-1	65/20	27	>70	NLP	NLP	Yes	1	1	None
V-2	63/22	69	47	NLP	LP	Yes	1	1	Trabeculectomy and Trabeculoplasty
V-7	29/29	25	26	20/40	20/30	Yes	0.6	0.5	None
V-8	25/19	8	50	NLP	NLP	Yes	1	1	Trabeculectomy
V-9	53/31	42	44	NLP	NLP	Yes	1	NA	Trabeculectomy
V-11	54/47	35	20	NLP	NLP	Yes	1	1	None
V-14	38/35	51	55	LP	NLP	Yes	0.8	0.6	Trabeculectomy
VI-3	32/18	54	20	LP	20/40	Yes	1	0.5	Trabeculectomy and Trabeculoplasty
VI-5	35/34	23	24	20/40	20/30	Yes	0.5	0.4	None
VI-6	33/33	23	25	20/30	20/30	Yes	0.6	0.6	None
VI-7	29/25	31	26	20/40	20/100	Yes	0.8	0.7	Trabeculectomy

Eight patients in Family A and nine patients in Family B had highly elevated intraocular pressure, completely cupped optic nerve, and complete blindness. Five patients in Family A and five patients in Family B had elevated intraocular pressures and increased cup-disc ratio of the optic nerve. All patients had visual field loss. Note: LP, light perception; NLP, no light perception; NA, not available.

### DNA sequence analysis

Nine genes, including *MYOC*, *CYP1B1*, *WDR36*, *OPTN*, and five genes within the interval (*ASB3*, *GPR75*, *CHAC2*, *RPS27A*, and *CCDC88A*) were sequenced using primers designed to amplify the complete coding regions and intron splice sites. PCR products were purified using QIAquik Gel Exaction Kit (Qiagen, Valencia, CA) and sequenced by both forward and reverse primers. The sequencing was performed using Big Dye ® Terminator v3.1 cycle sequencing kits (ABI, Foster City, CA) and was analyzed on the ABI 3100 Genetic Analyzer.

**Figure 2:**
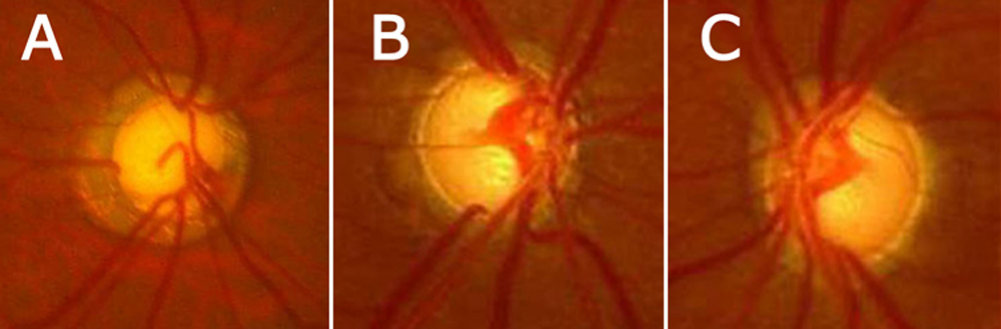
Fundus pictures of affected patients showed that the cup/disc ratios of the affected patients were very high. **A**: The picture displays the left optic nerve head of subject IV:1 in Family A. **B** and **C**: The last two pictures presents the left (**B**) and right (**C**) optic nerve head of subject V:14 of Family B. Note nerve fiber layer defect, notching, and excavation in all three fundus pictures. The disc asymmetry was shown between the two eyes of V:14 in Family B (**B**, **C**).

## Results

### Clinical features

Eighteen family members were studied in Family A including 13 affected and 5 unaffected individuals. Twenty-five family members were studied in Family B including 14 affected and 11 unaffected individuals (Figure 1). Age at examination ranged from 14 to 70 years old in Family A and ranged from 25 to 83 years old in Family B. The age of onset of disease ranged from 14 to 50 years old in Family A and from 18 to 47 years old in Family B. The detailed clinical features of the affected patients are listed in Table 1. Eight patients in Family A and nine patients in Family B had highly elevated intraocular pressure, completely cupped optic nerve, and complete blindness (no light perception [NLP] or light perception [LP]), which is diagnostic of end stage POAG (Figure 2, Table 1). Five patients in Family A and five patients in Family B had elevated intraocular pressures and increased cup-disc ratio of the optic nerve (Figure 2, Table 1).

**Figure 3:**
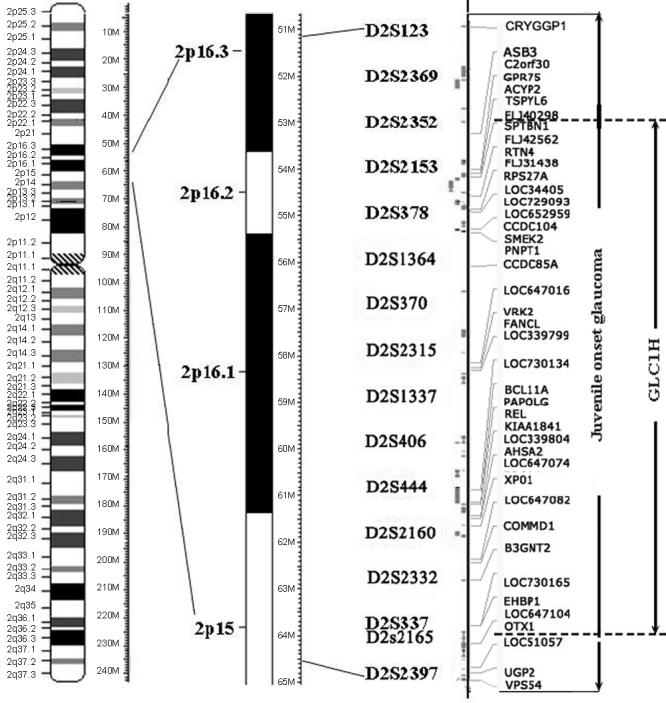
Chromosome 2 map. The order of short repeat polymorphism from the p15–16 region of chromosome 2 is shown. The interval of the two juvenile-onset Chinese families contains 42 genes/transcripts and is compared with the interval of GLC1H.

### Linkage analysis and haplotype analysis

STR markers, GLC1A (*MYOC*; 1q23), GLC1J (9q22), GLC1K (20q12), 5q22.1-q32, and 15q22-q24, encompassing previously known loci related to juvenile-onset POAG, were genotyped first. No linkage was found. Then, adult onset POAG loci, including GLC3B (2p36), GLC3A (2p21, *CYP1B1*), GLC1B (2cent-2q13), GLC1C (3q21–24), RIEG1 (4q25, *PITX2*), GLC1G (5q22, *WDR36*), IRID1 (6p25, *FOXC1*), GLC1F (7q35), GPDS1 (7q35-q36), GLC1D (8q23), Nail patella syndrome (NPS; 9q34, *LMX1B*), GLC1E (10p15-p14, *OPTN*), NNO1 (11p), AN2 (11p13, *PAX6*), VMD2 (11q12), MFRP (11q23), RIEG2 (13q14), GLC1L (15q11-q13), were genotyped; no linkage was found to any of these loci. No mutations were found by direct sequencing in the coding regions of any of the known glaucoma genes including *MYOC*, *CYP1B1*, *WDR36*, and *OPTN*. A whole genome scan using ABI Linkage Mapping Set v2.5 was performed using Family A. The scan revealed positive linkage to 2p15–16 with an LOD score of 5.01 at D2S337. Additional STR makers were genotyped, and the disease interval was narrowed down to 9.2 cM between D2S123 and D2S2397 using refining STR markers and haplotype analysis. Subsequently, genotype and linkage analysis was performed for Family B in this region. We found that Family B also showed complete linkage to the same region with the same (9.2 cM) interval between D2S123 and D2S2397. A maximum two-point LOD score of 5.01 was obtained at θ=0 for D2S337 in Family A and 6.30 at θ=0 for D2S378 in Family B. On the basis of allele size and haplotype analysis, the two families share the same disease haplotype, suggesting they have inherited the same mutation from a common founder. The maximum LOD scores were 8.93 at θ=0 for D2S378 and 9.9 at θ=0 for D2S337 when the two families were combined for analysis. The disease interval was defined to 9.2 cM between D2S123 and D2S2397 in these two Chinese families (Figure 1 and Figure 3). Table 2 shows the two-point LOD scores of the STR markers around the linkage site.

### Sequence analysis

There were 42 known or predicted genes within the interval (NCBI). No disease-causing mutation was identified in any of the five genes in this interval (*ASB3, GPR75, CHAC2, RPS27A*, and *CCDC88A*) after sequencing analysis.

**Table 2:** Two-point LOD scores between short tandem repeats and disease phenotype.

**STR marker**	**Genetic position (cM)**	**Physical position (Mb)**	**Family A**				
			**θ=0.0**	**θ=0.1**	**θ=0.2**	**θ=0.3**	**θ=0.4**
D2S391	70.31	46.26	−5.41	1.00	0.99	0.7	0.28
D2S123	73.61	51.14	−0.66	1.62	1.19	0.68	0.22
D2S2369	73.61	53.08	4.18	3.41	2.57	1.66	0.73
D2S2352	76.34	53.73	3.18	2.57	1.91	1.21	0.5
D2S378	77.43	57.18	2.63	2.10	1.53	0.94	0.37
D2S337	80.69	61.52	5.01	4.12	3.14	2.08	0.96
D2S2397	82.82	64.49	−10.03	−1.23	−0.54	−0.24	−0.08
D2S380	83.88	65.5	−3.34	3.24	2.57	1.70	0.77
**STR marker**	**Genetic position (cM)**	**Physical position (Mb)**	**Family B**				
			**θ=0.0**	**θ=0.1**	**θ=0.2**	**θ=0.3**	**θ=0.4**
D2S391	70.31	46.26	1.13	3.96	3.19	2.18	1.06
D2S123	73.61	51.14	−20.47	1.67	2.01	1.61	0.87
D2S2369	73.61	53.08	4.58	3.79	2.87	1.87	0.84
D2S2352	76.34	53.73	2.99	2.62	2.04	1.38	0.69
D2S378	77.43	57.18	6.3	5.23	4.04	2.73	1.32
D2S337	80.69	61.52	4.89	4.55	3.57	2.39	1.13
D2S2397	82.82	64.49	1.21	1.15	0.9	0.58	0.24
D2S380	83.88	65.5	−0.72	2.07	1.76	1.18	0.52

The disease interval for family A is between D2S123 and D2S2397 with the maximum LOD score of 5.01 for D2S337 at θ=0, and the disease interval for family B is between D2S123 and D2S380 with the maximum LOD score of 4.89 for D2S337 at θ=0.

## Discussion

Twenty-five loci have been associated with glaucoma [8,11], and four genes have been identified for monogenic glaucoma with Mendelian inheritance patterns including *MYOC, CYP1B1, OPTN*, and *WDR36*. *MYOC* is responsible for about 36% of juvenile-onset POAG cases and 2%–4% of adult-onset POAG cases [4,17,18]. *CYP1B1* is responsible for recessive congenital glaucoma [19]. *OPTN* is mainly responsible for normal tension glaucoma [20]. *WDR36* is responsible for adult-onset and low-tension glaucoma and accounts for 5%–17% of adult-onset POAG cases [7,8,21].

Here, we describe a new juvenile-onset POAG locus on 2p15-p16 linked to two large Chinese families. Interestingly, this early-onset POAG locus partially overlapped with an adult-onset POAG locus (GLC1H) reported recently [8] (Figure 3). This locus is next to a previously described adult-onset locus on chromosome 2 between D2S441 and D2S2232 [22], suggesting the possibility of the same gene causing both early-onset and adult-onset glaucoma as *MYOC* does [4,17,23]. The interval of GLC1H is 8.3 Mb between D2S2352 and D2S2165, which is within our interval [8].

An investigation of monogenic glaucoma may help elucidate the pathogenic mechanisms of late-onset complex glaucoma, which involves multiple genes and environmental factors. Complex glaucoma affects many more people, and no major gene has yet been identified for complex primary glaucoma. However, recently the *LOXL1* gene has been shown to be associated with exfoliation glaucoma (XFG), a secondary glaucoma to exfoliation syndrome (XFS) [24-26]. Because juvenile-onset glaucoma, which is associated with single-gene mutations, shares clinical and histopathologic features with adult-onset glaucoma, monogenic glaucoma genes like *MYOC* and *WDR36* may be associated with complex glaucoma. Identification of the disease-causing gene(s) in this 2p15-p16 locus linked to both early-onset and late-onset glaucoma has the possibility of finding a common pathway to these diseases and defining the underlying pathophysiology. Additionally, gene discovery may lead to an early DNA-based diagnosis test, which may contribute to therapeutic interventions at early stages of the disease and preserve vision.

## References

[1] Quigley HA (1996). Number of people with glaucoma worldwide.. Br J Ophthalmol.

[2] Ritch R, Shields MB, Krupin T. Classifications and mechanisms of the glaucomas. In: Ritch R, Shields MB, Krupin T, editors. The Glaucomas. Vol. II. St. Louis: Mosby; 1996. p. 717–725.

[3] Sheffield VC, Stone EM, Alward WL, Drack AV (1993). Johnson At, Streb LM, Nichols BE. Genetic linkage of familial open angle glaucoma to chromosome 1q21-q31.. Nat Genet.

[4] Stone EM, Fingert JH, Alward WL, Nguyen TD, Polansky JR, Sunden SL, Nishimura D, Clark AF, Nystuen A, Nichols BE, Mackey DA, Ritch R, Kalenak JW, Craven ER, Sheffield VC (1997). Identification of a gene that causes primary open angle glaucoma.. Science.

[5] Alward WL, Fingert JH, Coote MA, Johnson AT, Lerner SF, Junqua D, Durcan FJ, McCartney PJ, Mackey DA, Sheffield VC, Stone EM (1998). Clinical features associated with mutations in the chromosome 1 open-angle glaucoma gene (GLC1A).. N Engl J Med.

[6] Trifan OC, Traboulsi EI, Stoilova D, Alozie I, Nguyen R, Raja S, Sarfarazi M (1998). A third locus (GLC1D) for adult-onset primary open-angle glaucoma maps to the 8q23 region.. Am J Ophthalmol.

[7] Monemi S (2005). spaeth G, DaSilva A, Popinchalk S, Ilitchev E, Liebmann J, Ritch R, Heon E, Crick RP, Child A, Sarfarazi M. Identification of a novel adult-onset primary open-angle glaucoma (POAG) gene on 5q22.1.. Hum Mol Genet.

[8] Suriyapperuma SP, Child A, Desai T, Brice G, Kerr A, Crick RP, Sarfarazi M (2007). A new locus (GLC1H) for adult-onset primary open-angle glaucoma maps to the 2p15-p16 region.. Arch Ophthalmol.

[9] Wiggs JL, Lynch S, Ynagi S, Maselli M, Auguste J, Del Bono EA, Olson LM, Haines JL (2004). A genomewide scan identifies novel early-onset primary open-angle glaucoma loci on 9q22 and 20p12.. Am J Hum Genet.

[10] Sheffield VC, Stone EM, Alward WL, Drack AV, Johnson AT, Streb LM, Nichols BE (1993). Genetic linkage of familial open angle glaucoma to chromosome 1q21-q31.. Nat Genet.

[11] Wiggs JL (2007). Genetic etiologies of glaucoma.. Arch Ophthalmol.

[12] Pang CP, Fan BJ, Canlas O, Wang DY, Dubois S, Tam PO (2006). Lam Ds, Raymond V, Ritch R. A genome-wide scan maps a novel juvenile-onset primary open angle glaucoma locus to chromosome 5q.. Mol Vis.

[13] Wang DY, Fan BJ, Chua JK, Tam PO, Leung CK, Lam DS, Pang CP (2006). A genome-wide scan maps a novel juvenile-onset primary open-angle glaucoma locus to 15q.. Invest Ophthalmol Vis Sci.

[14] Lathrop GM, Lalouel JM, Julier C, Ott J (1984). Strategies for multilocus linkage analysis in humans.. Proc Natl Acad Sci USA.

[15] Lathrop GM, Lalouel JM (1985). Julier c, Ott J. Multilocus linkage analysis in humans: detection of linkage and estimation of recombination.. Am J Hum Genet.

[16] Oetting WS, Lee HK, Flanders DJ, Wiesner GL, Sellers TA, King RA (1995). Linkage analysis with multiplexed short tandem repeat polymorphisms using infrared fluorescence and M13 tailed primers.. Genomics.

[17] Shimizu S, Lichter PR, Johnson AT, Zhou Z, Higashi M, Gottfredsdottir M, Othman M, Moroi SE, Rozsa FW, Schertzer RM, Clarke MS, Schartz AL, Downs CA, Vollrath D, Richards JE (2000). Age-dependent prevalence of mutations at the GLC1A locus in primary open-angle glaucoma.. Am J Ophthalmol.

[18] Fingert JH, Heon E, Liebmann JM, Yamamoto T, Craig JE, Rait J, Kawase K, Hoh ST, Buys YM, Dickinson J, Hockey RR, Williams-Lyn D, Trope G, Kitazawa Y, Ritch R, Mackey DA, Alward WL, Sheffield VC, Stone EM (1999). Analysis of myocilin mutations in 1703 glaucoma patients from five different populations.. Hum Mol Genet.

[19] Stoilov I, Akarsu AN, Sarfarazi M (1997). Identification of three different truncating mutations in cytochrome P4501B1 (CYP1B1) as the principal cause of primary congenital glaucoma (Buphthalmos) in families linked to the GLC3A locus on chromosome 2p21.. Hum Mol Genet.

[20] Rezaie T, Child A, Hitchings R, Brice G, Miller L, Coca-Prados M, Heon E, Krupin T, Ritch R, Kreutzer D, Crick RP, Sarfarazi M (2002). Adult-onset primary open-angle glaucoma caused by mutations in optineurin.. Science.

[21] Hauser MA, Allingham RR, Linkroum K, Wang J, LaRocque-Abramson K, Figueiredo D, Santiago-Turla C, del Bono EA, Haines JL, Pericak-Vance MA, Wiggs JL (2006). Distribution of WDR36 DNA sequence variants in patients with primary open-angle glaucoma.. Invest Ophthalmol Vis Sci.

[22] Wiggs JL, Allingham RR, Hossain A, Kern J, Auguste J, DelBono EA, Broomer B, Graham FL, Hauser M, Pericak-Vance M, Haines JL (2000). Genome-wide scan for adult onset primary open angle glaucoma.. Hum Mol Genet.

[23] Fingert JH, Heon E, Liebmann JM, Yamamoto T, Craig JE, Rait J, Kawase K, Hoh ST, Buys YM, Dickinson J, Hockey RR, Williams-Lyn D, Trope G, Kitazawa Y, Ritch R, Mackey DA, Alward WL, Sheffield VC, Stone EM (1999). Analysis of myocilin mutations in 1703 glaucoma patients from five different populations.. Hum Mol Genet.

[24] Thorleifsson G, Magnusson KP, Sulem P, Walters GB, Gudbjartsson DF, Stefansson H, Jonsson T, Jonasdottir A, Jonasdottir A, Stefansdottir G, Masson G, Hardarson GA, Petursson H, Arnarsson A, Motallebipour M, Wallerman O, Wadelius C, Gulcher JR, Thorsteinsdottir U, Kong A, Jonasson F, Stefansson K (2007). Common sequence variants in the LOXL1 gene confer susceptibility to exfoliation glaucoma.. Science.

[25] Fingert JH, Alward WL, Kwon YH, Wang K, Streb LM, Sheffield VC, Stone EM (2007). LOXL1 Mutations Are Associated with Exfoliation Syndrome in Patients from the Midwestern United States.. Am J Ophthalmol.

[26] Hewitt AW, Sharma S, Burdon KP, Wang JJ, Baird PN, Dimasi DP, Mackey DA, Mitchell P, Craig JE (2007). Ancestral LOXL1 variants are associated with pseudoexfoliation in Caucasian Australians but with markedly lower penetrance than in Nordic people.. Hum Mol Genet.

